# Cyclopædia of Practical Surgery

**Published:** 1841-07-01

**Authors:** 


THE CYCLOPÆDIA OF PRACTICAL SURGERY. Edited by *Wm. B. Costello*, M.D. Illustrated with numerous Engravings and Wood-cuts.

SINCE our last notice of this work, two additional Parts have appeared, and the first volume, containing nearly 900 pages, has been completed. After the long suspension of its progress, which had necessarily awakened fears as to its continuance, it is satisfactory to see it now advancing at a steady pace, and maintaining the interest which its early parts had created. The editor has had to bear blame, perhaps unjustly, for not having kept time with the public—there must in such cases, be some scape-goat—but after all, the real delinquents are those who get off scot-free; we mean the contributors, who feel no compunction whatever for breach of promise to the editor, who is thus compelled in a work alphabetically arranged to disappoint his readers. Delay or disappointment in an undertaking of this magnitude is always detrimental, and should be avoided, if possible; a serious responsibility therefore rests with those, be they few or many, who mar the efforts of their fellow-labourers, by not completing at the appointed time the tasks they had undertaken, and no excellence of performance will be a sufficient compensation to them or to the public for such culpable remissness. The editorial duties, when conscientiously discharged, as they have been in the present instance, are sufficiently painful and laborious of themselves, and ought not to be aggravated by the failures of contributors on the one hand, which lead necessarily to the discontenting and estranging of patrons on the other.

The work itself has not disappointed the expectations of the profession. It is not made up of a series of dissertions, with here and there one by an able hand, shedding its brilliancy and light over a dull blank of monotonous mediocrity—an oasis in the waste. There is an uniformity, a substance, a completeness, a power and tone throughout, creditable not alone to the individual writers, but to the time and country in which we live. The Cyclopædia of Practical Surgery may not only challenge, but will advantageously sustain comparison with any other similar work of other countries, and if we infer the extent and superiority of the education of the surgeons of this country as a body, from the high character of the work before us, considering it as a book of reference prepared for them expressly, we cannot but rejoice at the changes which have been brought about within the last quarter of a century.

Part VII. commences with the continuation of Cancer, an article so ably drawn up as fully to justify, in our opinion, the high eulogy of Dr. Warren, of Boston, " the ablest treatise on cancer that ever was written." We shall not, however, re-open this subject. Its author, Dr. Walshe, has begun to reap his laurels, having, within a few weeks, been appointed to the chair of Dr. Carswell, in University College.

The article Cancrum Oris, by Dr. Hennis Green, is well and lucidly drawn up. We must however content ourselves with passing praise, as the abundance of the matter contained in these two parts, and their claim upon our notice, bear an overpowering proportion to our limited space.

Next, is an article on Castration by Sir Astley Cooper. It is simply a description of the operation, and the circumstances under which it is performed. It possesses, in addition to the interest attaching to anything bearing the recommendation of so revered a name, that of being the last contribution of his pen. It may be here stated as a trait characteristic of his ardour and zeal in the cause of science, that he evinced the deepest interest in the progress of the Cyclopædia of Surgery, and at the time he was seized with his last illness, was actually engaged in re-writing his Memoir on Dislocations, for this work.

The article Cataract, by Dr. Watson, is an excellent contribution, and is illustrated with a well-executed engraving of the various forms of the disease, and of the instruments now generally in use for performing the different operations of cataract. The subject of the appreciation of the operations themselves in regard to the preference which one may deserve over any other in its applicability to a particular case, is well handled. We, however, must pass this over, contenting ourselves with one extract embracing some novel points in the diagnosis.

"*Catoptrical exploration of the state of the lens as regards its transparency or non-transparency.*—This mode of exploring the eye, first invented by Professor Purkinje of Breslaw, has come into general notice only since M. Sanson published his clinical observations on the subject. 'The young practitioner ought never to pronounce absolutely even on the existence of cataract without dilating the pupil by *belladonna*, and examining the eye *catoptrically*; and the most experienced may derive advantage from exposing in this way the whole field of the disease to his view, and testing the state of the crystalline.'*

If a lighted candle be held before the pupil of a sound eye, three reflected images of it are seen situated one behind the other. Of these the anterior and posterior are erect, the middle one inverted. The anterior is the brightest and most distinct, the posterior the least so. The middle one is the smallest.

If the candle be moved, the two erect images follow it; but the inverted image moves in the opposite direction, though not so quickly and extensively as the other two.

The anterior erect image is produced by the cornea; the posterior, by the anterior surface of the lens; and the middle or inverted image is from the posterior surface.

If the whole crystalline body be opaque, no image but the anterior erect one from the cornea is seen. This is of course the case also if the anterior part alone be opaque; but if it is the centre or the posterior part only which is opaque, the two erect images are seen, but the middle or inverted one is not.

To apply this catoptrical method to assist the diagnosis of cataract, when the ordinary mode of examination may not have cleared away all doubt, 'the observer and the patient should be placed in moderate day-light; the patient's back is to be turned towards the window; he should be seated, so that the observer may look rather down into the eye than upwards: and a candle is to be used which burns steadily, and does not blaze much.

Cataract, even at an early stage, obliterates the inverted image, and renders the deep erect one very indistinct. Glaucoma, only when much advanced, obliterates the inverted image, while, in all its stages, it renders the deep erect one more evident than it is in the healthy eye.'

Dr. Mackenzie has found that in glaucoma, at a middle stage, the inverted

---

* Mackenzie, p. 642.

image is pretty distinct, when formed near the edge of the crystalline; but if the candle be brought in front of the eye, the inverted image is less distinct, and in some cases is altogether extinguished. This extinction of the inverted image is owing to a loss of transparency in the kernel of the lens which, in glaucoma, Dr. M. has found, suffers a peculiar degeneration, characterized by dryness of substance, and a reddish brown colour.

In incipient lenticular cataract the inverted image, though changed neither in colour nor in size, is indistinct, and its outline as if washed off. It is extinguished long before the cataract is fully developed—a fact of the greatest importance, Dr. M. remarks, in the diagnosis betwixt glaucoma and cataract, because in lenticular cataract, it is not the kernel of the lens, but the superficial laminæ, which are first affected, so that the formation of the inverted image by any part of the posterior surface of the crystalline body is prevented.

'In capsulo-lenticular cataract, the inverted image fades much sooner than in mere lenticular cataract; and even when the capsule, or the superficial substance of the lens, seems to be alone opaque, the inverted image disappears much sooner than we should expect from the apparently moderate degree of opacity.

'In lenticular cataract, there is merely a general reflection, but no distinct image, from the anterior surface of the crystalline body.

'If the lens is not in its place, but has been absorbed in consequence of an injury, been removed by an operation, or fallen down into a dissolved vitreous humour, neither inverted nor deep erect image is formed.

'In the diagnosis of incipient cataract and incipient amaurosis, the catoptrical test is perfectly decisive; for in amaurosis, uncombined with glaucoma, the three images are always distinct, while in even the early stage of cataract, the inverted image is obscure. The diagnosis of incipient cataract and incipient glaucoma requires the catoptrical test to be familiar to the observer, else he may not be able to distinguish, that when the candle is held in the axis of the eye, the inverted image is indistinct in both diseases, but whenever it is moved to one side, it becomes distinct in glaucoma, and remains obscure in cataract."*

To this succeeds an article on Catheterism from the pen of the editor of the Cyclopædia, and which being, as our French neighbours would say, a subject within his own speciality, claims our notice. After disposing of the definition of the word catheterism, he thus addresses himself to his subject.

"However inconsistent the avowal may appear with our present purpose, it is nevertheless perfectly true, that dexterity and suavity in the introduction of instruments is less to be acquired by the precepts hitherto inculcated in books, than by practice and example, and even some of the rules laid down to guide us on this point do not deserve to be particularly extolled. The surgical doctrine of catheterism stands in great need of simplification, and the few rules that really are useful with relation to it require to be redeemed from that over refined elaborateness, the effect of which is too frequently to intimidate the young surgeon, thus insuring a clumsy and painful performance of this surgical ministration, from the mere dread of a barely possible mischief. To him who possesses the necessary anatomical and physiological knowledge, sounding the bladder when the urethra is healthy and unobstructed may soon be rendered a simple and easy operation. But the instructions derived from books alone will not suffice; they are, in too many instances, the offspring of superficial observation or vague theory, and too frequently when obstacles occur, a reference to those rules will afford no assistance whatsoever in overcoming them.

---

* Mackenzie, p. 640, 1.

The great diversity of conformation in the parts concerned in catheterism, the variety of instruments employed for the purpose, with many other circumstances, seem to authorize the multiplication of rules to govern us in its performance. But the real truth is, the attempt to regulate and refine on all the incidents belonging to it, by obscuring the simple nature of the subject, has only tended to perplex and embarrass us in the consideration of it. Had the canal of the urethra been of equal diameter or purely cylindrical, and had it followed the same direction throughout its entire length, the operation of passing the catheter would be comparatively simple, and one general rule would embody all that could be said on the subject. But such is not the case. The points, therefore, that occur in its course where the cylindrical form does not exist, and those in which its direction is altered, are those which it is important to study, with a view to the easy, painless, and safe mode of introducing the catheter." 735.

In his reasoning on the condition of the urethra, Dr. Costello makes use of its natural division into a *fixed* or post-pubic, and a *moveable* or ante-pubic portion; the former commencing at the triangular ligament, and terminating in the bladder, the latter extending from the glans to the triangular ligament. On this division, he founds his rules for the introduction of instruments of every kind. He thus expounds this point:—

"In our consideration of the urethra it will be seen that we consider the entire canal in man as composed of a male and female portion; the former associated with the penis, moving with it in every direction, serving for the object of sexual congress specifically, and but incidentally only for conveying the urine out of its reservoir; in a word, the *moveable* division of the urethra; the latter, similar in structure in both sexes, in both having the same length and direction, and more or less closely attached to bones or enveloped by muscles, the *fixed*, or female urethra. Philosophically speaking, the female urethra is the type of the canal and exists alike in both sexes; all the ante-pubic portion is but a prolongation of the tube, superadded foı a purpose wholly different from that of the excretion of urine. All this anterior portion is confessedly moveable, and the admission is equivalent to a demonstration of the inutility of the rules laid down for the introduction of instruments, as far as this portion of the canal is concerned, whether it be by the ordinary mode *above the pubis*, or by the so pompously named *tour de maître*. An instrument, whether straight or curved, can be carried down to the bulb, no matter whether the direction of this portion of the canal be upwards, downwards, forwards, or lateral; whether it be curved or straight. This point being indisputable, the question arises,—what mode of penetration is easiest for the operator and for the patient? what mode should be preferred with reference to the fair direction of the bec of the catheter into the commencement of the second division of the canal, and its ulterior progress through this fixed portion towards the bladder? The best, the easiest, the least painful is undoubtedly that which insures the unimpeded passage of the bec throughout the whole course from the glans to the bulb; which leaves no doubt as to the bec arriving at this point without stopping short of it or going beyond it, and which places it most favourably by the performance of a very simple manœuvre, for falling directly into the opening of the triangular ligament." 740.

After finding fault with the position which by former rules the surgeon is directed to take in regard to the patient upon whom he is about to introduce the catheter, he thus describes his own mode:—

"The patient being in the recumbent posture, the surgeon stands on his right. The relaxation of the parts having been procured in the manner already described, he seizes the penis behind the glans, between the ring and middle finger, while the index finger and thumb, set on opposite sides of the glans, uncover the orifice of the urethra. The glans being inclined towards the right thigh, the surgeon, holding the catheter like a writing pen, in an arch over the right thigh, presents its bec to the orifice of the glans, which is already turned to meet it. Throughout the whole course which the catheter has now to run, its bec glides upon the right side of the canal, the concavity of its curve looking to the right side, while the curve itself is seen arching the canal above the scrotum, until the point arrives at the bulb.

The ease, brilliancy, and lightness of execution of catheterism depends entirely upon the manœuvre now to be performed, of passing from the bulb to the entrance of the muscular portion,—the sill, as it were, that divides the whole urethra into its male and female portions. The shaft of the catheter is now resting over the thigh, but not in contact with it, and its bec is in the bulb.

It must be lifted from the thigh, and brought up to the perpendicular by a movement of a quarter of a circle, the centre of which is the bulb; in other words, the handle of the catheter moves round a quarter of the base of an inverted cone, while the bec pivots on its apex. But these movements, though described as distinct, must be made to follow each other without interruption; they must not be executed, as if they were two 'times' of the same process, but must run into and blend finely with each other, so that the movement of lowering, in which they terminate,—that movement in which the handle of the sound is brought down from the perpendicular till it lies inclined between the thighs, shall either bring the bec exactly into line with the muscular part of the urethra, when by a gentle impulsion applied at this part of the movement, it is propelled directly into it, or toppling on the right edge of the aperture in the triangular ligament, as it were on the edge of a bowl, it falls into it.

There will of course be some variation of the process, where the patient is sounded in the erect posture. In such case, the surgeon may prefer introducing the catheter in a line with the hollow of the groin, instead of arching it over the thigh; but the variation will merely consist in the instrument describing a lesser fraction of the circle, while the hand is bringing it up from the groin to the perpendicular." 741.

This article on a subject of so much importance abounds with clear practical views and rules which cannot fail to improve our acquaintance with it, while the Essay itself must enhance the reputation of its author. We shall make room but for one more extract, descriptive of Dr. Costello's *self-guiding* catheter, which is now becoming better known to the profession.

"The catheters already in use being commonly of equal diameter, and rounded or oval at the point, present the same unvarying relation to every point of the canal whether it be narrowed or dilated; and hence the obstacles (natural obstacles, as they are termed) that oppose the progress of the instrument upon the slightest misdirection of it in those points of the canal where its diameter varies abruptly. I have had a fair share of experience of this subject and have thought much upon it, and no doubt now remains on my mind of our possessing the power of obviating the difficulties of catheterism, as arising from these obstacles, by the form of the instrument which has now been for some time subjected to the test of daily use.

In speaking formerly of the choice of a catheter I dwelt much on the importance of its bulk; the largest, when not disproportioned to the calibre of the canal, being in general the best. The reason of the preference is obvious,—the large instrument, according as it advances in the urethra and unfolds the membrane, forms a small vacuum before its bec, and thus its route is opened and prepared for it in a manner which does not obtain when a small instrument is employed, for in the latter case the folds of the canal are in close contact with its point.

The qualities that seem to me desirable in the catheter are the following:—

First, the volume, or size, should be full, in proportion to the calibre of the urethra.

Secondly, its shape should be such that the small vacuum in advance of the bulk of the catheter, and produced by it, shall be occupied by a small guiding prolongation of its point, inclined to the upper surface of the canal.

Thirdly, on its under surface its shape, between the tip of the small prolongation and the part at which its fullest diameter is developed, shall be such as to occupy or accommodate itself to the depression as it passes them, and to depress and flatten the eminences on the under surface of the urethra as it passes over them.

Fourthly, that the eyes shall not be too large, and that they shall be so placed as not to present any obstacle to the progress of the sound, either by their being plugged or stopped up by the lining membrane of the urethra.

It will be evident on examining the catheter I propose, that its form fulfils all the conditions required in relation to the canal in its passage down to the bladder.

Its form in all respects is the same as that of an ordinary catheter, except from the eyes to the termination of the bec, and in this portion its shape is entirely different from that of the instrument in general use. The whole of this portion consists of three parts, a neck, head, and beak, and bears a strong resemblance to the neck, head, and bill of a swan.

When a catheter of this shape is passed into the urethra the bulk of the head is sufficient to unfold the membrane in advance of it; the bill occupies the vacant space thus made, and continues to move along smoothly in contact with the upper surface of the urethra, from which its extreme point is so fashioned as to incline slightly towards the axis of the urethra. When it arrives at the bulb, the bulk of the head occupies this depression, while the guide or bill is necessarily directed into the middle of the isthmus, or commencement of the *fixed* portion of the canal; the handle being now gently lowered, the bulk of the head ascends out of the depression of the bulb, and favoured by the inclined plane, in which the head and bill are blended together, glides imperceptibly over the sill formed by the under edge of the triangular ligament. It moves on freely through the muscular portion till it reaches the prostate; here again, the guide, keeping close by the upper surface, overtops the eminence of the verumontanum, and depressing it, leads the head of the catheter gently over it. The bill then enters the bladder at the upper part of its orifice, making way for the head of the catheter as it had done throughout the whole of the passage. To resume: the chief and valuable peculiarity of this catheter consists in the fidelity with which its upper surface keeps to the upper surface of the urethra, while the bulk of the body, tapering upwards to the end of the bill, successively unfolds the depressions and climbs upon the eminences which it meets in its course along the inferior surface of the canal." 745.

Caustics and Cauterization the contributions of Mr. Ure, and Cephalæmatoma, that of Dr. Walshe, we pass over, not from any disregard of their respective merits, which are considerable, but from a wish to introduce a new subject of interest to the reader. Chondritis, a new denomination for the ulceration of cartilage, attests the progress of pathology in a branch that has been cultivated with the most gratifying success in this country; and chiefly by Sir B. Brodie. The recent experiments also of Mr. Liston, which are alluded to in the present article, afford strong confirmation of the views of the pathology of the ulceration of cartilage propounded in the Cyclopædia in the articles ARTICULATION and CHONDRITIS. This part of the subject was so fully treated under the former head by Mr. *Blackburn*, that Mr. *Spencer Wells*, the author of Chondritis, has merely added the confirmatory experiments of Mr. Liston, enlarging chiefly upon the more practical part of the subject. He, however, adds an interesting observation in connexion with a disease as yet but little known, the puerperal form of arthritis. He says, "I had lately an opportunity of examining the body of a female who died about a fortnight after delivery, with symptoms which were ascribed to puerperal peritonitis. There were, however, no traces of abdominal disease. While examining the organs of the pelvis, our attention was directed to the left sacro-iliac synchondrosis by an appearance of bulging in that situation. We found that the cartilages covering the opposed surfaces of the ilium and sacrum had been completely absorbed, and the bones were consequently denuded. There was a large quantity of pus contained between and around the bones, and their abraded surface was covered by an exudation of recent lymph."

A very graphic description of the symptoms usually observed in the different classes of cases in which ulceration of cartilage is a prominent symptom, on the principal and primary disease, is given, and Mr. *Wells* proceeds to the diagnosis of the chronic cases from other diseases, and this part of the subject is so well treated, and of such practical importance, that we give the section on diagnosis entire. The diseases for which ulceration of cartilage may be mistaken are "inflammation of the synovial membrane; articular osteitis; scrophulous deposit in the articular extremities of the bones; the peculiar change of structure in the synovial membrane described by Sir Benjamin Brodie; inflammation of the ligaments; external chronic abscess; inflammation and effusion into a bursal sac; and neuralgia."

"The diagnosis of ulceration of cartilage from inflammation of the synovial membrane is not difficult. In synovitis the pain is at first severe, but gradually diminishes; in ulceration, on the contrary, it is as a general rule at first slight, gradually increasing, and is aggravated in a far greater degree by any approximation of the articular extremities of the bones. In synovitis the swelling occurs simultaneously with, or very soon after the first attack of pain: in ulceration patients generally suffer very severe pain for weeks before any swelling is discoverable. In synovitis the form of the swelling is characteristic, from the bulging of the fluid at those parts of the joint unprotected by ligaments: in ulceration the swelling has the form of the healthy joint. In synovitis there is fluctuation throughout the disease: in ulceration there is no fluctuation till suppuration has taken place.

Caries of the head of the bones is marked by its slow progress, and the earlier occurrence of swelling. In the scrophulous cases there is very little pain, and far more swelling than in the case of disease of the cartilage; there are other marks of a scrophulous diathesis; the disease rarely occurs after puberty; the swelling does not take the form of the joint, but gives an appearance of enlargement of the head of the bone, so peculiar, that those who have observed it once, will not fail to recognize it. Suppuration, when it comes on, is not confined to a single collection of matter, but forms a succession of abscesses.

In the words of Sir Benjamin Brodie, the gelatinous degeneration of the synovial membrane is marked by 'the gradual progress of the enlargement and stiffness of the joint without pain, and the soft elastic swelling without fluctuation, in the majority of cases, enable us to distinguish it readily from all the other morbid affections to which the joints are liable.'*

Inflammation of ligaments is marked by the superficial character of the pain and its increase, not by all motions of the joint, but only by those which put the affected ligaments on the stretch. This is characteristic. The absence of the other symptoms of ulceration of cartilage will also be remarked.

The circumscribed nature of the swelling which results from bursal inflammation, its chronic character, the absence of pain, together with the occupation of the patient, will be sufficient to detect the nature of the disease in its early stages on the most cursory glance. But if suppuration have taken place, the sac have ulcerated, and pus has been discharged into the cellular tissue around the joint, a mistake may be made. In order to show that this error is not an imaginary one, I may state, that I knew of a limb which was condemned to amputation, though this was the sole disease. The diagnosis is, of course, most simple, especially if the disease occur in the knee or elbow, as is almost invariably the case. If the pus were within the joint, it would be internal to the patella or olecranon, whereas in the other case it is external. The other marks of disease of the cartilage are also wanting.

To those who have not seen much of articular diseases, it may appear unlikely that mere chronic inflammation of the soft parts around a joint, attended by suppuration, should be mistaken for serious disease of the joint itself. Such, however, is not unfrequently the case; and sometimes the error is very far from a gross one, though an attentive enquiry into the history and symptoms would suffice to establish the diagnosis. Thus, if we compare the symptoms with those of ulceration of cartilage, we find that evident tumefaction is one of the earliest signs of chronic inflammation of the soft parts; there is also more stiffness of the joint in the early stage: the pain is more confined in its situation; aggravated in a less degree by motion of the joint, but in a greater by superficial pressure over the seat of pain: it does not increase to the same severe extent, nor is it accompanied by the startings of the limb: it is not so much relieved by rest. It will be remembered also that generally speaking it is some months before suppuration takes place from cartilaginous disease.

Sir Benjamin Brodie states that a large majority of the cases of suspected diseased joints which occur in fashionable practice are nothing more than neuralgic affections. The same assertion may be made with perfect accuracy in the case of certain unfortunate females, who frequently fall under the observation of the general practitioner. Their irregular habits of life; the mental distress many of them suffer; the state of nervous excitement common to the order; and its increase by means of ardent spirits in the large majority, induce a state of system which predisposes to diseases of the same character as those which result from more refined dissipation. The most severe pain is felt in the neighbourhood of some joint, and as this is for some time unaccompanied by other symptoms, it is more likely to be mistaken for ulceration of cartilage than any thing else. The diagnosis, however, is not difficult. The general appearance of the patient is that of an hysteric nervously excitable person—her mode of life is probably such as tends to exhaust or cause irregular distribution of nervous influence—probably there is neuralgia in other parts,—but even suppose, as is sometimes the case, the patient appears florid and healthy, even then the symptoms are characteristic. The pain at first comes on in paroxysms, the intermissions being perfect; it is evidently seated in the integuments; there is extreme tenderness on the slightest touch, while on continuing the pressure, considerable force may be exerted, and the pain be thus removed. The pain is not confined to the joint, and remains

---

* "Pathological and Surgical Observations on the Diseases of the Joints, p. 86."

for a considerable time without other evidence of local disease, and when swelling does come on, it is a diffused œdema. Frequently extensive motion of the joint can be borne without pain. These marks are quite sufficient to establish our diagnosis, but we must avoid the other extreme, and not be led by the presence of neuralgia to infer the absence of organic disease. As far as my personal observation has gone, I should be led to doubt the position that simple morbid irritability of any tissue can continue for any considerable length of time, without inducing increased arterial action." 770.

The morbid appearances in the different articulations are then succinctly described, and Mr. *Wells* proceeds to the subject of treatment, paying a well-merited tribute to the genius and industry of Sir Benjamin Brodie.

"The indications for the local treatment of a joint, the cartilages of which are ulcerated, are to prevent any increase of the morbid action from motion, shocks, or any other cause of irritation. Then regarding the ulcerative process as the result of chronic inflammatory action to lessen and subdue this by means of counter-irritation." 771.

With regard to the best means for keeping the joint in a state of absolute quietude, Mr. *Wells* dilates at some length upon the advantages of starched bandages and other immoveable apparatus, by means of which, he says, "we have an apparatus easily procured, readily applied, and as speedily removed: a gentle and uniform compression is exerted on the limb, the joint is kept immoveably fixed, and a patient who, under other circumstances, would be confined to a sick chamber, is enabled to walk in the open air with the assistance of crutches, and as the bandages are very light, and adapt themselves to every irregularity of the surface of the limb, the joint may be kept in any position that is desired."

Of the means employed to subdue the morbid action going on within the joint, directions are given for the application of leeches and cupping, and with regard to counter-irritation, we cannot do better than quote the valuable observations of Mr. Wells.

"In children, blisters kept open with some irritating dressing, afford much relief to the pain, and if the limb be kept quiet, this may be all that is necessary; but in adults, issues made with caustic potash, or the potassa cum calce, are far more serviceable. Sir Benjamin Brodie says they have a greater effect over ulceration of cartilage than any other disease of joints. They should be made as near the joint as is compatible with the safety of the synovial membrane. In some cases the first application is followed by an immediate cessation of the symptoms; in almost all, considerable relief is afforded, and if quiet be maintained for some weeks, the cure is often completed. Sir B. Brodie remarks, that the benefit derived from the issue does not appear to be in proportion to the quantity of pus discharged from its surface, and he thinks it is a decidedly better plan to keep the issue open by rubbing it two or three times a week with caustic potash, or sulphate of copper, than to employ beans. When these issues are applied, however, in combination with the ordinary methods of fixing the joint, their good effects are often destroyed by the involuntary motions of the limb. It is evident that the effects are likely to be far more beneficial when the joint is kept perfectly immoveable, and to effect this after applying the starched bandage, I have removed a piece about the size of half a crown, and applied the potassa cum calce to the skin at this spot. In this way perfect rest and the benefit of the issue were simultaneously and easily obtained.

The application of moxæ, and the formation of eschars by means of heated irons have been much recommended, and were formerly in pretty general use in articular diseases, but they have of late years been superseded by caustic issues and setons. Most patients have a great horror of the actual cautery, and the difficulty of regulating the extent of their action is anothei strong objection to all the usual modes of cauterization. Instances are on record in which the synovial membrane was penetrated, and the most deplorable results followed. The evils, however, which arise from the abuse of a remedy, are not arguments against its proper use, and I believe that moxæ, when properly applied, are more manageable and efficacious than any other form of counter-irritation. The simplest and best with which I am acquainted, is made with paper which has been steeped in the liquor plumbi diacetatis, and afterwards dried. This is folded into a narrow strip, and rolled up after the manner of a bandage, till it gives a surface about the size of a sixpence. It is held on the point of skin where we wish to make the eschar by a pair of forceps or scissors, and a light applied. By blowing on it, it burns brightly like tinder without flame, and produces an eschar of sufficient extent which separates in a day or two. They may be applied in this way in conjunction with the starched bandages, by cutting out a small piece of the latter, and thus all the benefits of counter-irritation may be secured, with perfect quietude of the limb. In Paris, agaric has been lately used to form moxæ, and it answers the purpose very well. Severinus and Rust are the principle advocates for the actual cautery. The latter author is very minute in his directions as to the mode and extent of its application, but it appears unnecessary to describe his practice, as all the benefits may be derived from the moxæ I have described, without any of the ill effects which have been observed to follow the hot iron." 773.

The treatment after suppuration has taken place is then discussed, together with the general treatment, but we have quoted so largely that we have only space for one remark strongly illustrative of the benefit of a knowledge of medicine to the surgeon, and another proof that, to be a good surgeon a man must be a good physician also.

"Before amputation be decided on, the lungs should be carefully examined, for I have now seen three instances in which very extensive pulmonary disease existed in cases of diseased joints in scrofulous patients (and which, it was thought, had commenced in ulceration of cartilage,) the general symptoms of pulmonary disease being singularly latent. There was in fact nothing to induce a supposition of chest affection, while very extensive disease was evident on auscultation. Amputation in such cases would be useless torture, and the surgeon would have the credit of hastening his patient's death. I believe many of the cases we used to hear of, where disease was said to commence in the lungs as soon as the diseased joint is removed, are to be accounted for in this way: the lungs being diseased, but the symptoms rendered latent by the action going on in the joint. On the other hand, if the lungs be sound, and there is no hope of saving the joint, the sooner it is removed the better, as the local irritation, if long protracted, will almost certainly be a cause of disease in some vital organ."

We consider this article on the whole just what a Cyclopædia article should be—a condensed account of previous knowledge on the subject, with interesting observations deduced from the experience of the writer. It has a striking contrast with the prosy Germaniform effusions of many of the aspirants for literary renown in the present day, and we would advise them to take a hint from this and other articles by the same writer, and, rejecting transcendental speculations, confine themselves to a simple exposition of what they know of their subject, and above all confine their remarks to the object of the work in which they appear.

We pass over several articles of merit to notice one on Club Foot by Mr. H. T. Chapman, in which the subject is handled with clearness and impartiality.

"The investigation and establishment," says Mr. Chapman, "by Dr. Stromeyer of Hanover, of the correct ætiology of club-foot, his successful revival of the operation of Thilenius for its cure, and the practical bearing of the same principles on other defects and distortions of the frame, have given this subject a greater importance in the eyes of the profession, than almost any recent improvement in surgery, and have excited very general surprise that the treatment of these deformities should have been so long abandoned to the mere mechanic. A review of the opinions entertained by successive writers upon this branch of surgery, will show that scarcely any addition to our knowledge of the nature and treatment of these affections was made from the time of Hippocrates to the latter part of the last century (1784), when Lorenz, a surgeon at Frankfort, divided the tendo achillis for the cure of a case of club-foot, under the direction of Thilenius." 781.

"Stromeyer's immediate predecessors, particularly Scarpa and Delpech, had thrown much light upon the nature of these affections; but sufficient obscurity still remained to check the repetition by others of Delpech's not very successful experiments. An investigation into the influence of the respiratory system of nerves in producing disorder of certain muscles of the trunk, and thus occasioning curvature of the spine, led Dr. Stromeyer through the secondary to the primary sources of club-foot, exposed its real nature, and enabled him to lay down the true system of treating it with so much certainty and amplitude, that succeeding writers have done little more than confirm the principles, for the development of which we are indebted to him." 782.

This point is resumed in the discussion of the opinions of different physiologists, as to the causes of club-foot, in a quotation from Dr. Little.

"'Rudolphi gave a more complete view of the origin than any of his predecessors, and one more consistent both with physiology and pathology. He concluded that congenital talipes and the analagous deformity of the hand (club-hand) arise from disordered influence of nerves on muscles in the fœtal state, by which their contraction is prematurely excited, and often in so vehement a manner, that the mother experiences pain from the convulsive motions; the limbs thus become distorted, and permanent deformity is often thereby occasioned. He observes, that the authors who have maintained the injurious influence of external and mechanical causes, such as pressure of the uterus through an improper position of the fœtus, appear to have been unaware, that these distortions are not unfrequently witnessed in embryos of three or four months. Rudolphi not only points out the influence exercised on the muscles of the extremities by powerful and general causes affecting the entire nervous system of the fœtus, but states that talipes often occurs in infants, in other respects well-formed, from simple spasm.' To Delpech, however, belongs the merit of having first worked out and practically applied the correct principle of treating these affections." 784.

Dr. Little's own opinions on the subject are moreover quoted *in extenso*, the result being thus summed up by Mr. C.

"We may assume it, therefore, as fairly established, that genuine club-foot, in all its varieties, is produced by irregular action of certain muscles, which irregular action consists in a disturbance of the balance existing naturally between the antagonising systems, the flexors and extensors, the adductors, and abductors of the foot; that this disturbance may occur in two ways; one system of muscles may be altogether deprived of nervous influence—may be paralysed, in short, and incapable of counter-balancing their antagonists in a state of *ordinary* action; or, retaining their ordinary power, the same set of muscles may be overcome by their antagonists preternaturally excited—in a state of *extraordinary* or spasmodic action; and, finally, that the occasional co-existence with congenital talipes of congenital club-hand, squinting, stammering, and spasmodic contractions throughout the muscular system, and the occurrence of club-foot as a consequence of convulsions during dentition, of fever, measles, croup, &c. in infants, of apoplexy,—or even of hysteria, in adults,—all indicate an origin dependent upon imperfect development or lesion, upon changes either of structure or function in the cerebro-spinal system, with scarcely less certainty than that with which many instances of acquired club-foot may be traced to direct local injury or irritation of a particular nerve." 786.

The article is well illustrated with woodcuts representing the distorted limbs, as well as the apparatus employed. The essential nature of the operation consists in a puncture of the skin followed by a subcutaneous division of the tendon. "By far the majority of operators," says Mr. C., "have adopted the views and follow the practice of Stromeyer, the various improvements suggested having reference to the form of knife, the distance from the heel at which section should be performed, and the mode of dividing the tendon, whether from behind forwards, or the reverse, whether by a transverse or oblique section. 'There has indeed,' as Dr. Little remarks, 'been no improvement of the principles laid down by Delpech, the value of which was first satisfactorily demonstrated and practically illustrated and enforced by Stromeyer. But there has been a departure from these principles, which Stromeyer has justly designated a retrogade process.'"

"Whenever the section of other tendons besides the achilles tendon is required, it is better to divide the tendons of those muscles which produce the inversion or eversion first; and, having brought the foot in line with the leg, that is, having reduced the deformity to the state of talipes equinus, then to operate upon the tendo achillis.

Not unfrequently before the contraction can be fully overcome, a transverse section of the plantar fascia is called for." 793.

We could have wished to dwell at some length on an article on Congelation, by Dr. A. Buchanan of Glasgow, but our space forbids this. It bears the impress of careful observation and a philosophic mind.

We however pass over this article on Congelation, as well as two or three others in this part, with the less regret, as we propose to return to them in a future number. Amongst these we may mention Condyloma by Henry James Johnson, and Cystitis by the Editor himself.

The next article we shall dwell on is one on Cornea by Mr. Wharton Jones. Mr. Jones thinks that the staphyloma corneæ is not, as commonly supposed, a protrusion of the cornea become opaque with the iris adhering to its posterior surface; but actually inodular tissue, which has been formed on the anterior surface of the iris, which has protruded in consequence of destruction of the cornea, partial or complete. The following extracts develops this view in detail.

"If in scrophulous, catarrhal, or catarrho-rheumatic ophthalmia, there be a penetrating ulcer of the cornea, the aqueous humour, as has been already mentioned, escapes, the iris falls forward into contact with the cornea, and a small part of it is perhaps prolapsed through the ulcerated opening. The progress of the ulceration being stopped by the yielding of the inflammation, the prolapsed portion of the iris, and the ulcerated part of the cornea are involved in one cicatrice. The opening in the cornea being thus closed, the aqueous humour again collects, and the anterior chamber is restored; though somewhat diminished, in censequence of the partial adhesion between the iris and cornea (*synechia anterior*). There is no prominent distention on the front of the eye in this case, because, as the inflammation subsides, the small protruded portion of iris shrinks and flattens; but if the destruction of the cornea has gone on farther, either by extension of ulceration from a continuance of the inflammation, or by the giving way of an abscess of the cornea, and considerably more of the iris has protruded, the prolapsed portion of the iris does not shrink when the inflammation begins to abate, as in the former case, but remains, and forms a projection at the part of the cornea implicated, which is generally the lower or lateral. This projection is at first merely a bag of the iris distended by the aqueous humour, and is called *staphyloma iridis;* but, by and by, its exposed surface becomes covered by an opaque firm tissue, of the nature of the *tissue of cicatrice*, and this tissue is incorporated at the base of the tumour with the sound cornea. The projection, the mode of origin of which I have just described, is a *partial staphyloma;* it is not a distention of the cornea itself, but a protruded portion of the iris covered by a *new tissue,* intended to supply the loss of substance which the cornea has sustained. The mode of origin of a *total staphyloma* is essentially the same, but differs only in degree. The whole or greater part of the cornea being destroyed, as occurs in gonorrhœal, purulent, and very often in variolous ophthalmia, as also that of new-born infants, the whole iris falls forward, the pupil becomes closed, and the aqueous humour, being thus allowed to accumulate in the posterior chamber, the iris is kept distended in the form of a tumour on the front of the eye. Its surface gradually gets covered with an opaque cicatrice-like tissue, or pseudo-cornea, which assumes a greater or less degree of thickness, and a total staphyloma is the result. Sometimes the central part only of the cornea is destroyed, a ring of the circumference still remaining; the staphylomatous projection has then the form of a small globe stuck on the front of a larger, but if disease has extended to the ciliary body, the whole front of the eye is prominent like a blunt cone." 844.

Founded on this particular view of the pathology of the disease, Mr. W. Jones's prophylactic treatment is peculiar and deserves mention.

"When an inflammation of the eye has run so disastrous a course, that the conditions for the formation of a total staphyloma are laid, any treatment which may be adopted can have for its object, not to save the eye as an organ of vision, but to prevent it from degenerating into a tumour, which not only causes great deformity, but is a source of considerable irritation even to the opposite eye, so much so, that the patient seeks for its removal by operation, sooner or later.

*Prophylactic Treatment.*—According to the account of the mode of formation of total staphyloma above given, it appears that the supply of aqueous humour in the still-existing posterior chamber, is what keeps up the distention of the iris, so that the pseudo-cornea which is moulded on its surface, presents the form of a round prominence on the front of the eye. If this be the case, the destruction of the source of the aqueous humour by breaking in upon the integrity of the posterior chamber, is the means which offers to prevent the development of the staphylomatous projection. The simplest plan of effecting this appearing to me to be the extraction of the lens, I put the operation into practice in the following case:—

A man, about twenty-two years old, came to me labouring under the effects of severe purulent ophthalmia of both eyes. In the right eye, the cornea being destroyed and the pupil closed, the iris protruded and was distended with aqueous humour. The left eye had also suffered very much; there was penetrating ulcer, prolapsus iridis, and consequently considerable distortion and contraction of the pupil. Both eyes were still affected with the inflammation, and it was very doubtful whether the left eye could be prevented from getting worse, especially as it was evidently kept in a state of additional irritation from the presence of the staphyloma in the right. By an incision with a Beer's cataract-knife through the protruding and distended iris, the lens was extracted. Severe re-action followed; less perhaps in consequence of the operation, than from the patient not being in a situation to take proper care of himself. The iris did not again become distended; on the contrary, the eye shrunk, and irritation being thus removed, the left eye progressively recovered, as far as the organic changes it had already undergone allowed, and further than there had been reason to hope for, as sufficient vision was preserved to enable the patient to resume his employment as a porter.

In those cases in which the eye is destroyed by purulent ophthalmia, whether in adults or new-born infants, by gonorrhœal ophthalmia, variolous ophthalmia, &c. and in which staphyloma does not result, but the cicatrice which forms in the place of the cornea is flat and the eyeball becomes atrophic, I suspect the lens has escaped on the giving way of the cornea.

A fully formed total spherical staphyloma is a source of great deformity; its removal therefore is often sought for in order that an artificial eye may be worn. But what principally demands its removal, sooner or later, is the irritation which it keeps up, and which is apt to be communicated to the opposite eye." 845.

This article is illustrated by a well-executed plate, containing eight very expressive figures.

We now come to the article Diplopia, the last in Part VIII. As double vision with two eyes is merely a disturbance of the conditions on which single vision with two eyes depends, Mr. Jones commences his article with an enquiry into those conditions.

" An affection of the retina is the primary condition of a visual perception, but a visual perception has in itself nothing to do with the optical apparatus regulating the transmission of the light which produces the affection. The question, therefore, why certain affections of the two retinæ should yield but a single, and others a double visual perception, is strictly physiological. All the purely physical explanations which have been offered of binocular vision have obviously reference alone to conditions not at all connected with the visual perception, but, from the nature of the optical apparatus in front of the retinæ, necessary, in order that the images of objects may be simultaneously projected on particular parts of those nervous expansions.

The particular parts of the two retinæ on which the rays of light from the same point of an object simultaneously fall, when the eyes have their natural corresponding direction, are the point of either membrane corresponding to the axis of the eyeball, and the various points or papillæ of the temporal half of one, and of the nasal half of the other, similarly situated in reference to it. These points or papillæ, called *corresponding*, *identical*, or *analogous*, have naturally, it is said, such a sympathy, that the simultaneous affection of them is followed by a single visual perception only: whilst double vision results from simultaneous impressions on any other parts of the two retinæ.

Objections have been urged against this doctrine of corresponding points of the retinæ. Mr. Wheatstone's ingenious observations are quite decisive in exposing the exclusiveness of the doctrine as above stated, but that they do not overthrow it altogether, will I think be made to appear in the course of this article." 873.

From the enquiry which follows, Mr. Jones sums up thus—

"When from any cause there is a loss of the natural correspondence of the optic axes, the parts of the two retinæ on which the images of the same object are simultaneously projected are not corresponding parts; therefore, in accordance with what has been above said, the sensations arising from the two impressions are separately perceived by the mind, and the consequence is double vision. Double vision with two eyes is thus in itself not a disease, but, from the organization of the eyes, a natural result of derangement of those conditions on which single vision depends. The proximate cause of the derangement alluded to is most frequently irregular or impeded action of the muscles of the eyeball; but it may be an organic contraction of parts, some morbid production in the orbit, or the like, displacing the eyeball.

The two images in diplopia, are often distinguished into true and false—or real and imaginary; but such a distinction is improper, as the one image, although it may be less distinct, is not more false or imaginary than the other, both being equally the result of sensation produced by the impression of rays of light on the retinæ. That one of the two images is more distinct than the other, is owing to the circumstance that in one eye the impression is made on the central part of the retina, which is more sensible than any other; while in the other eye, the impression falls on a part of the retina, which according to the degree of deviation, is more or less distant from the centre. The adjustment of that eye, moreover, which receives the impression on the centre of its retina, corresponds more with the distance of the object looked at; the relative position of the two images depends upon the direction of the deviation of the eyes. As sometimes the deviation of the axes of the eyes exists only when the person looks in particular directions, and at certain distances, so does the double vision in such cases take place only when the patient looks in those directions, and at those distances.

The deviation of the optic axes giving rise to double vision may exist in various degrees, from an evident squint to a scarcely perceptible cast. It is in this latter case that the double vision is most marked, as the two retinal sensations are about equal in force, in consequence of the images of the object being projected on or near the centres of the retinæ, and on parts of it not far from being corresponding. When the deviation of the optic axes is still slighter, the two retinal images contend as it were to attract the mind's attention, and the object appears as if oscillating with velocity before the eyes, the consequence of which is confusion of perception when the two eyes are employed, and sometimes vertigo. As the person in this state can see things distinctly only when he closes one eye, the affection has been called *Monoblepsis*.

The name *diplopia* is usually confined to those cases in which the misdirection of the eye is so slight as scarcely to attract notice, but in which, for the reason above explained, the double vision is strongly experienced by the patient.

The irregular or impeded action of the muscles of the eyeball, giving rise to diplopia, may be owing to an affection of the muscles themselves or of their nerves, or it may be owing to disease or injury of the brain, or to drunkenness or fear, or to derangement of the *primæ viæ*, &c. But this is not the place to discuss the various primary affections on which the derangement of the muscles of the eye depends. It is enough to explain the nature of diplopia with two eyes, in order that, as a symptom in any particular disease, it may be appreciated at its due value." 178.

The article concludes with a practical remark of so much interest and value as to be well worth quoting. "In most presbyopic persons Dr. Arnott has ascertained double vision in either eye singly to exist in a slight degree, and he has discovered that it may be entirely obviated by having the glasses of the spectacles so placed, that the axes of the eyes may fall upon them more or less obliquely. Vision is at the same time rendered very much more distinct."

The Cyclopædia of Surgery conducted to its conclusion in the spirit and style of the present volume, cannot fail of the object which it seems to aim at above all others, and which is, to form a work of reference in which the present state of surgery shall be fully and clearly expounded. The earnestness of its editor to carry out this object will moreover obtain for him pardon for past interruptions, if he continue to pursue an onward course with any tolerable degree of steadiness. This first volume stamps a high character on the work, and regularity of issue of the future parts, is all that is required to secure for it extensive patronage. In closing our remarks for the present, we should not omit to state, that our estimate of the Cyclopædia of Surgery is fully confirmed by the critiques of the Continental Reviewers.

